# A comprehensive benchmark of tools for efficient genomic interval querying

**DOI:** 10.1093/bib/bbaf379

**Published:** 2025-07-29

**Authors:** Richard A Schäfer, Rendong Yang

**Affiliations:** Department of Urology, Northwestern University Feinberg School of Medicine, Chicago, IL 60611, United States; Department of Urology, Northwestern University Feinberg School of Medicine, Chicago, IL 60611, United States; Robert H. Lurie Comprehensive Cancer Center, Northwestern University Feinberg School of Medicine, Chicago, IL 60611, United States

**Keywords:** interval, genomics, query, data

## Abstract

Efficiently querying genomic intervals is fundamental to modern bioinformatics, enabling researchers to extract and analyze specific regions from large genomic datasets. While various tools have been developed for this purpose, there lacks a comprehensive comparison of their performance, memory usage, and practical utility. We present a systematic evaluation of genomic interval query tools using simulated datasets of varying sizes. Our benchmarking framework, segmeter, assesses both basic and complex interval queries, examining runtime performance, memory efficiency, and query precision across different tools. This comprehensive analysis provides insights into the strengths and limitations of different approaches to genomic interval querying, offering guidance for tool selection based on specific use cases and data requirements. The segmeter framework and all benchmark data are freely available, facilitating reproducibility and enabling researchers to conduct their own comparative analyses.

## Introduction

Efficiently querying specific genomic regions is fundamental in bioinformatics, enabling researchers to extract relevant information from large genomic datasets. These files may contain up to billions of records, making data retrieval computationally expensive. Querying by genomic regions allows researchers to focus on specific regions of interest, such as protein-coding regions to characterize the consequences of genetic variants on the proteome [1, 2]. In addition, genomic queries are integral to numerous algorithms that assign biological data to genomic feature elements. For example, querying genomic regions is crucial in peak calling to identify enriched regions linked to functional elements [3], in homology detection to find conserved sequences across species [4–6], and in RNA-RNA interaction prediction to pinpoint binding sites within specific gene regions, such as UTRs [7]. Naive approaches to query intervals line by line become inefficient as the data volume grows. To facilitate rapid retrieval of relevant data from large datasets, specialized tools have been developed to allow users to focus on specific regions of interest. Tools for querying genomic intervals can be broadly categorized based on how they structure data for efficient retrieval. While some methods explicitely generate external index files to accelerate lookups, others dynamically construct in-memory data structures. [8] introduced tabix, a tool that applies the concept of indexing to position-sorted and compressed tab-delimited formats. By generating an index, tabix enables fast, region-based queries, significantly improving the efficiency of working with large genomic datasets and extending the indexing approach used in alignment data formats like BAM to general intervals. Other tools have been developed to support the querying of genomic regions, such as bedtools [9] and BEDops [10]. bedtools is a versatile suite of utilities that allows the manipulation and analysis of genomic intervals in BED, GFF, VCF, and other file formats. It incorporates a hierarchical indexing scheme used in the UCSC genome browser to internally index genome coordinate ranges [11–13]. In that regard, the UCSC provides command-line utilities for interval data manipulation [14]. BEDops, another powerful tool, specializes in optimizing the performance of operations on BED files. It requires the interval data to be sorted, which allows the processing of intervals sequentially rather than loading the interval data into memory, thereby reducing runtime and computational requirements. Similarly, gia [15] is designed to stream sorted interval data but also supports operations on unsorted data, by which the data is first loaded into memory. An interval is a tree data structure that can hold a set of intervals and allows efficient operations on intervals. The intervaltree library has been widely used can be incorporated in analyses. In bedtk [16], implicit interval trees are utilized to overlap lists of intervals. Rather than storing explicit pointers between nodes, implicit interval trees maintain a binary search tree using array indices to represent parent-child relationships. This reduces the memory overhead substantially, but preserves efficient interval query capabilities. Based on this concept, the COITrees library (https://github.com/dcjones/coitrees) improves the query performance by storing the nodes in a van Emde Boas layout [17].

This library is utilized in granges [19], providing genomic interval manipulation capabilities comparable to bedtools. In GIGGLE [20], the intervals are stored in a B+ tree to allow large-scale interval comparisons. Other data structures that can be used for querying intervals overlaps include the nested containment list [22] and the augmented interval list (AIList) [18]. In this work, we benchmarked available command-line tools for querying genomic intervals, evaluating their runtime performance and memory requirements, storage and general usability.

## Materials and methods

**Figure 1:**
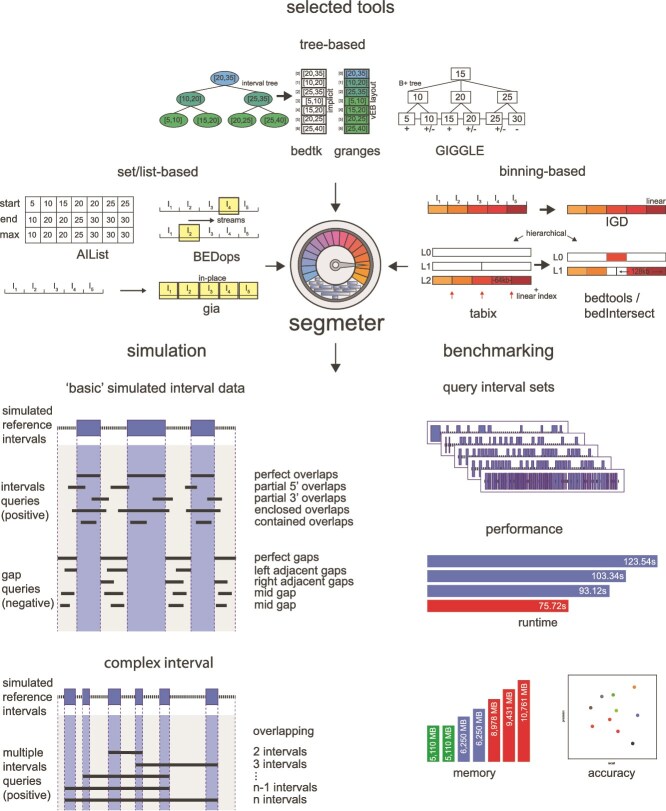
Overview of the segmeter framework, the benchmarked methods and the data generated.

We systematically compared the performance of various methods for searching interval data (Table 1). For that, we created an integrative benchmarking framework termed segmeter that is freely available at https://github.com/ylab-hi/segmeter. Figure 1 provides an overview of the framework and illustrates the modes of operation: data simulation (mode sim) and benchmarking (mode bench).

**Table 1:** Tools capable for querying genomic intervals

Tool	Data structure	Indexing^a^	Data sorting required	Data format	Compression	Reference
AIList	Augmented interval list	$\times $	$\times $	BED	$\times $	[18]
BEDops	Flat interval set	$\times $	✓	BED	.starch	[10]
bedtools	Hierarchical binning	$\times $	$\times $	BED, GFF, VCF	.gz	[9]
bedtk	Implicit interval tree	$\times $	$\times $	BED	.gz	[16]
gia	Flat interval set	$\times $	$\times $	BED	.gz,.bgz	[15]
granges	Cache oblivious interval tree	$\times $	$\times $	BED	.gz,,.bgz	[19]
GIGGLE	B+ tree	✓	✓	BED, VCF	.bgz	[20]
IGD	Linear binning	✓	$\times $	BED	.gz	[21]
tabix	Binning and linear index	✓	✓	BED, GTF VCF (others)	.gz,.bgz	[8]
UCSC utils	Hierarchical binning	$\times $	$\times $	BED	$\times $	[14]

$^{a}$ Indexing refers to the tool creating a separate index.

### Data simulation mode

In the data simulation mode, artificial intervals are generated randomly and placed on chromosomes with customizable properties. These include the number of intervals (option --intvlnums), the size of each interval (option --intvlsize, default: 100-10000), and the gap size between intervals (option --gapsize, default: 100--5000) which are generated within a user-defined minimum and maximum range. The intervals are placed sequentially on chromosomes, ensuring that most chromosomes have at least ten intervals do not exceed two billion in length. If all chromosomes become fully occupied, any remaining intervals are placed onto additional scaffolds. For each generated reference interval, segmeter produces ten corresponding interval and gap queries for benchmarking purposes, as illustrated in Fig. 1. Interval queries are designed to overlap the reference intervals in various ways. These include perfect overlaps, where the query interval is identical to the reference interval, partial overlaps at either the 5$^\prime $ or 3$^\prime $ ends, enclosing intervals that fully contain the reference interval, and contained intervals entirely within the reference interval. Gap queries, on the other hand, target the spaces between reference intervals and do not overlap the intervals themselves. These include perfect gaps, where the query matches the gap between two intervals exactly, gaps adjacent to the start or end of an interval, and two gaps entirely contained within larger gaps. In addition, segmeter subsamples the resulting query datasets by 10%, 20%,..., up to 100% of their original size. Similarly, the generated intervals are used for complex queries that cover multiple intervals. In particular, segmeter generates for $n$ intervals on each chromosome queries of different lengths, covering $2$ to $n$ intervals. These are distributed into bins corresponding to deciles.

### Benchmarking mode

In the benchmarking mode, segmeter evaluates each tool on simulated data (data simulation mode), or arbitrary query target pairs. In doing so, segmeter assesses the runtime for indexing (if required) and querying the intervals, the memory requirements, and precision of the query. Since the methods vary in functionality, we focused the benchmark specifically on detecting overlaps. In particular, the tools are supposed to return the actual target interval where the overlap with the queries occurred. Please refer to the supplementary information for more detailed information about how the different tools were executed. In segmeter, we included tabix, bedtools on unsorted interval data, bedtools on sorted interval data, bedtools using tabix for random access, bedops and bedmap from the BEDops utility, giggle, gia on unsorted and sorted data, bedtk on unsorted and sorted data, IGD, AIList, and bedIntersect from the UCSC utilities. Benchmarking was primarily performed on unsorted data. For tools requiring preprocessing steps, such as sorting or indexing of reference and query data, we included these computational costs in the indexing and querying runtime, respectively. Precision was determined based on the accuracy of returned overlaps. For basic interval queries, we assessed precision by verifying that the reported overlaps matched the reference intervals. For complex queries, we measured precision by calculating the distance in the number of overlapping intervals. In this mode, segmeter generates output files containing statistics for each benchmarked tool (option --tool).

## Annotate variants with regulatory elements

We retrieved whole-genome variant data and benchmarked annotation tools against tissue-specific regulatory regions. To enable efficient overlap analysis, we first converted the variant calls into BED format using the gvf2bed utility from the BEDops toolkit. The resulting BED files were then intersected with regulatory regions obtained from ENCODE (accession: ENCFF879ZPI), which includes putative cis-regulatory elements (cCREs) identified across multiple tissues. This comparison enabled us to assess the coverage and performance of annotation tools in capturing biologically relevant

### System environment

All benchmarks were conducted on a MacBook Pro (2023) with MacOS 15.2, equipped with an Apple M2 chip and 32 GB of unified memory. We used the Docker containers provided by segmeter the considered tools, which were restricted to a single CPU core and allocated a maximum of 12 GB of memory to ensure a fair and reproducible evaluation.

## Results

We used segmeter  v0.13.x to generate datasets with $10$, $100$, $1000$, $10\,000$, and $100\,000$ intervals. For that, we used the default values for --intvlsize and --gapsize. This results in 10 to 4287 intervals distributed on 24 chromosomes, with an average length of 5490 for each interval and 2591 for the gaps (see Supplementary Table S1, Figs S1 and S2). Subsequently, we evaluated each tool using segmeter in three different runs. The resulting metrics were evaluated in the following.

### Basic queries

**Figure 2:**
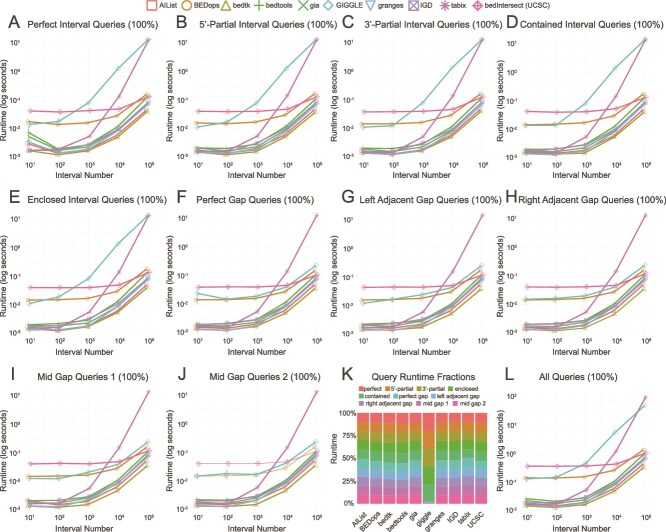
Runtime comparison using simulated interval data. Positive queries (A–E) overlap with reference intervals, while negative queries (F–J) target gaps between intervals. Panel (K) shows each query’s percentage of total runtime, while panel (L) displays the cumulative runtime.

We analyzed the runtime needed for interval and gap queries, with results shown in Fig. 2. Additionally, we measured the runtime of bedtools and bedtk on sorted data, bedtools combined with tabix for random access, and bedmap from the BEDops utility. However, as we account for sorting time, we did not observe any runtime improvements over unsorted data (see Supplementary Figs S3–S5). For overlapping interval queries (Fig. 2A–E), bedtk and AIList consistently demonstrated superior performance across all interval query sets, with granges, IGD, and gia exhibiting an increased runtime. tabix performed adequately for smaller query sets (up to 1000 queries) but showed poor scaling with increased query volume. While GIGGLE and UCSC utils showed comparable performance for smaller query sets, GIGGLE’s runtime degraded to match tabix at higher query volumes, while UCSC utils aligned with BEDops. Similarly, bedtools initially performs comparable to granges and IGD, but its runtime eventually matches that of UCSC utils and BEDops. In contrast, UCSC utils maintained linear scaling, performing better than BEDops with the number of interval queries increasing to $10^{5}$. For gap queries (Fig. 2F–J), which identify regions without overlap of the reference interval, performance patterns were similar with notable exceptions. GIGGLE showed improved performance, matching BEDops and UCSC utils, while tabix maintained its poor performance. In bedtk and granges, we observed an equivalent performance for small query sets which diverged at higher volumes. The runtime hierarchy remained consistent with bedtk leading, followed by AIList, while bedtools, granges, gia, and IGD continued to exhibit a slower runtime. Analysis of the average fraction of runtime spent on interval and gap queries (Fig. 2K) revealed consistent proportions across all tools except GIGGLE, where interval queries consumed a notably larger share of total runtime. The total runtime across all query types (Fig. 2L) showed bedtk maintaining the best performance throughout. For smaller query sets (up to 1000), granges and AIList performed similarly as second-best, but at higher volumes granges aligned with gia and IGD. Additionally, UCSC utils, bedtools, and BEDops formed a slower tier, while GIGGLE and tabix exhibited the highest runtimes overall.

### Complex queries

**Figure 3:**
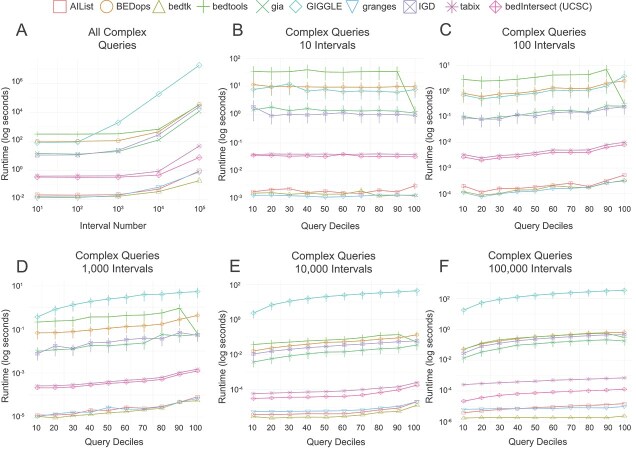
Runtime comparison using simulated complex interval data. Runtime for all complex queries over all interval datasets (A), and averaged runtime per interval query for datasets with $10$ (B), $100$ (C), $1000$ (D), $10\,000$ (E), and $100\,000$ intervals.

We analyzed the runtime performance of complex queries, as illustrated in Fig. 3. First, we summarized the overall runtime for all complex queries combined (Fig. 3A). The results indicate that AIList, bedtk, and granges form a group with consistently fast runtimes across all interval datasets. This is followed by tabix and UCSC utils, which exhibit moderate runtimes. Another distinct tier, with runtimes approximately ten times higher, includes gia, BEDops, IGD, and bedtools. Notably, GIGGLE demonstrates the highest runtime, which increases further as the set size of the interval increases. A similar pattern can be observed when inspecting the runtime for each dataset individually (Fig. 3B–F). For small datasets (Figs 3B-C), the runtime decreases when the query spans nearly all intervals (10th decile), while the runtime in larger datasets remains constant over different deciles (Fig. 3E–F). Interestingly, with an increasing number of reference intervals (Fig. 3D–F) we observe a decrease in the runtime per query.

### Memory requirements

**Figure 4:**
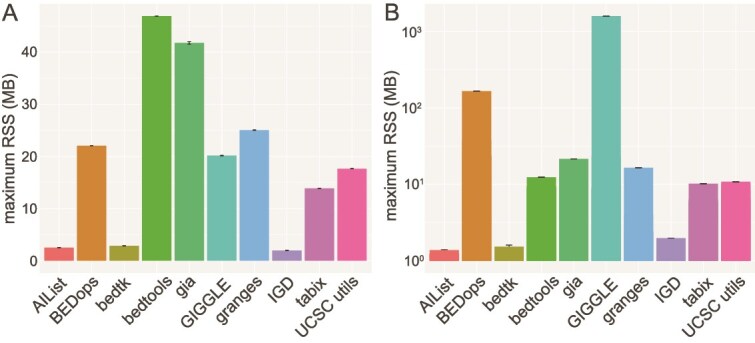
Peak memory usage over all interval datastes for basic (A) and complex queries (B).

We also analyzed the memory requirements of each tool when querying different interval datasets, as shown in Fig. 4. When performing all basic queries (Fig. 4A), AIList, bedtk, and IGD demonstrated the lowest memory usage. A second tier follows, consisting of tabix, UCSC utils, GIGGLE, BEDops, and granges, which exhibit moderate memory consumption. In contrast, gia and bedtools require approximately twice the memory of the second tier, placing them in the highest memory usage category. For complex queries (Fig. 4B), we observed distinct memory usage patterns across tools. AIList, bedtk, and IGD remained the most memory-efficient, while gia exhibited reduced memory consumption compared to its usage in basic queries, now aligning with tabix, UCSC utils, bedtools, and granges. Meanwhile, BEDops showed increase memory usage, which is only exceeded by GIGGLE.

### Indexed creation

**Figure 5:**
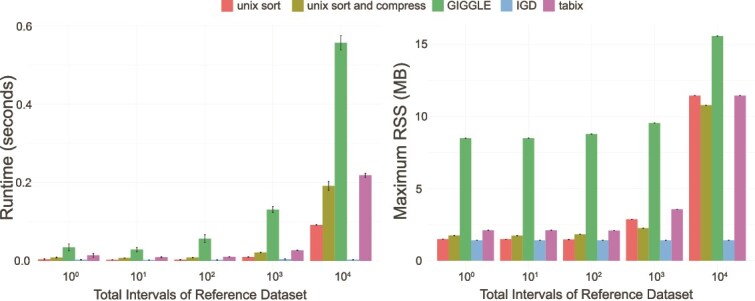
Memory requirements for index creation.

We analyzed the runtime required for indexing various tools, as shown in Fig. 5. While indexing is a one-time cost, it contributes to the overall runtime when querying intervals. In the selected tools in this benchmark, only GIGGLE, IGD, and tabix require a preprocessed index when querying interval. Other tools such as BEDops simply require the data to be sorted. We included simple sorting and sorting with compression. Our results show that GIGGLE exhibits the highest runtime and memory usage across all datasets. In contrast, IGD maintains a consistent runtime and memory footprint regardless of the dataset size. It can be seen that tabix compared to simple sorting only demonstrate comparable performance, though they experience a significant increase in runtime and memory usage under larger datasets.

### Accuracy

When the selected tools are benchmarked, segmeter also captures the precision of queries that overlap with reference intervals. We primarily used this for internal purposes to ensure that the tools function correctly and that all overlapping intervals can be retrieved. For that, segmeter is configured to process the output of a selection of tools, so that the results are comparable (see supplementary information). With the exception of granges, we encountered perfect precision when querying the tools with our generated datasets (see Table S4).

### Usability

Most tools could be easily installed using a designated package manager, such as bedtools and tabix via APT (https://salsa.debian.org/apt-team/apt), gia and granges via Cargo (https://github.com/rust-lang/cargo), while others, like AIList, BEDops, bedtk, IGD, and GIGGLE require compilation using Make. We made modifications to the data handling in GIGGLE, so it could run correctly (see supplementary information). In most tools, the functionality is restricted to simple overlapping queries, while BEDops, bedtools, and gia provide a comprehensive feature set.

### Benchmarking variant annotation

**Figure 6:**
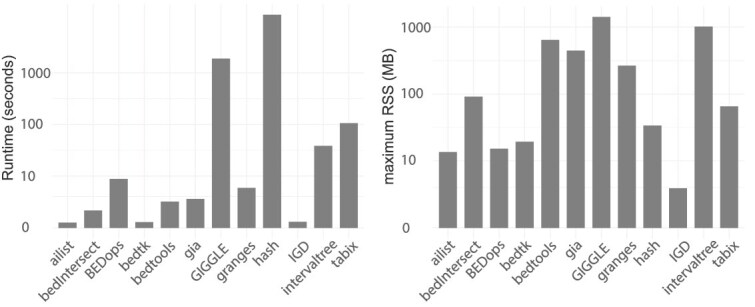
Time measurements and peak memory usage for variant annotation.

To evaluate the performance of tools for large-scale variant annotation, we benchmarked the runtime of ten widely used genomic interval query tools by intersecting whole-genome variant calls with ENCODE cCREs (accession: ENCFF879ZPI). We also included a naive hash table-based approach and the intervaltree Python library as baselines. The results reveal substantial variability in performance. The fastest tools completed the task almost instantaneously, while others required significantly more time—sometimes by orders of magnitude. A subset of tools, including ailist, bedtk, and IGD, demonstrated moderate performance, finishing within a reasonable timeframe. Conversely, tools like GIGGLE and the naive hash-based method exhibited the longest runtimes. Memory usage showed a similarly wide range. The maximum resident set size spanned from a few megabytes to several gigabytes. Notably, IGD was the most memory-efficient, requiring only a few megabytes. In contrast, tools employing tree-based data structures consistently consumed more memory, as shown in Fig. 6.

## Discussion

In this study, we evaluated the performance of various genomic interval query tools using simulated datasets ranging from $10$ to $100,000$ intervals, providing insights into the scalability and efficiency of the established methods. Although we observed a relationship between the tools and their data structures, other factors, such as implementation and data handling, may also play a role. This is beyond the scope of this benchmark. Our results showed that the implicit interval tree implementation in bedtk consistently demonstrated superior performance, followed by the augmented interval list approach in AIList. The efficiency of these data structures stems from their ability to balance memory efficiency and query speed—implicit interval trees eliminate pointer overhead while preserving efficient interval query capabilities, whereas augmented interval lists achieve fast lookups through strategic augmentation of interval endpoints. In contrast, tools employing more complex data structures, such as the B+ tree in GIGGLE, exhibited poorer scalability with larger datasets, suggesting that the overhead of maintaining these structures outweighs their theoretical advantages for genomic interval queries. The B+ tree structure, despite its higher maintenance overhead, can offer advantages in more complex scenarios such as pangenome analysis, in which multiple overlap relationships need to be handled. For complex queries spanning multiple intervals, we found that the choice of data structure became even more critical. Tools utilizing simpler, memory-efficient structures, such as bedtk or AIList, exhibited consistent performance across dataset sizes. In contrast, the performance tools with more elaborate indexing schemes diminished. This suggests that for genomic interval queries, the overhead of maintaining complex tree structures may not be justified by the performance benefits, particularly when dealing with large-scale data. Memory usage patterns also reflected the efficiency of different data structures. The low memory footprint of AIList, bedtk, and IGD demonstrates the advantages of their streamlined data representations. In contrast, tools like bedtools, which load more data into memory, showed higher memory requirements. This trade-off between memory usage and query performance appears to be a crucial consideration in tool design, with the most successful tools finding an optimal balance. In the indexing requirements, we observed that the B+ structure of GIGGLE has the highest indexing costs, while simpler indexing approaches such as in IGD demonstrated more practical trade-offs between index creation time and query performance. This suggests that for the considered genomic interval queries, complex indexing strategies may not provide sufficient performance benefits to justify their computational overhead. It remains to be investigated to what extent these methods can demonstrate their usefulness when handling larger interval queries and complex datasets. We found that tools requiring sorted input, such as BEDops to show competitive performance, but at the cost of preprocessing overhead. When this preprocessing is accounted for, we found no improvement to methods working on unsorted data. This highlights the importance of considering not just the query algorithm itself, but also the preprocessing requirements when evaluating tool efficiency. The precision analysis revealed that most tools achieved perfect accuracy, with granges being the notable exception. This suggests that while different data structures can significantly impact performance, they generally maintain accuracy in overlap detection. For general interval queries, our results suggest that simpler, memory-efficient data structures like implicit interval trees and augmented interval lists may be more practical than theoretically optimal but complex structures.

## Conclusion

In this work, we introduced segmeter, a comprehensive framework for benchmarking tools used in querying interval data. We evaluated widely used methods in the field, assessing their runtime, memory usage, and overall applicability. While a variety of data structures exist for genomic interval queries, our results suggest that simpler, memory-efficient structures often outperform more complex ones in practical applications. Among the tools tested, bedtk and AIList demonstrated the highest efficiency on large datasets, whereas others, such as GIGGLE and tabix, faced scalability challenges. This benchmark primarily assessed the tools themselves; however, their performance is also influenced by the characteristics of the interval data and the implementation details of the underlying data structure.

**Key Points**

- We introduce segmeter, a comprehensive benchmarking framework that generates artificial interval data and enables the systematic evaluation of genomic interval query tools.
- We conduct a systematic evaluation of ten widely-used genomic interval query tools, assessing their runtime efficiency, memory usage, and query precision across different simulated datasets
- Our evaluation reveals that for interval queries, lightweight data structures such as implicit interval trees—which store intervals in an array simulating a balanced tree—and augmented interval lists—which enhance sorted interval lists with metadata for faster pruning—consistently achieve better performance in terms of runtime and memory usage than more complex alternatives like pointer-based interval trees or nested containment lists.

## Supplementary Material

supplementaryinformation_bbaf379

TableS1_bbaf379

TableS2_bbaf379

TableS3_bbaf379

TableS4_bbaf379

## Data Availability

We have established a permanent data repository on Zenodo (DOI: https://doi.org/10.5281/zenodo.14880992), which contains the simulated data used in our benchmarking. We provide detailed statistics on the simulation data in Supplementary Table S1. Supplementary Table S2 contains all runtime measurements and memory usage for the different benchmark runs, while Supplementary Table S3 presents the same metrics for the index creation process. Finally, Supplementary Table S4 summarizes the precision results of the benchmarks. The repository includes all relevant scripts and configurations to reproduce our results.

## Code availability

We distribute segmeter under the MIT license and make it freely available through GitHub (https://github.com/ylab-hi/segmeter). The repository includes comprehensive documentation, installation instructions, and Docker containers to ensure reproducibility. All benchmarking scripts and configuration files used in this study are also included in the repository.
