# Supplementary material for: A comprehensive benchmark of tools for efficient genomic interval querying
Source: Brief Bioinform. 2025 Jul 29;26(4):bbaf379. doi: 10.1093/bib/bbaf379 (PMC12306436; doi:10.1093/bib/bbaf379)
Supplement: supplementaryinformation_bbaf379 [file supplementaryinformation_bbaf379.pdf]

PAPER

Supplementary Information: A Comprehensive Benchmark of Tools for Efficient Genomic Interval Querying

Richard A. Schäfer[1,*] and Rendong Yang[1,2,*]

[1]Department of Urology, Northwestern University Feinberg School of Medicine, Chicago, IL 60611, United States and [2]Robert H. Lurie Comprehensive Cancer Center, Northwestern University Feinberg School of Medicine, Chicago, IL 60611, United States
*Corresponding author. richard.schaefer@northwestern.edu, rendong.yang@northwestern.edu

Execution of external tools

The tools and their corresponding commands are listed below, as they were utilized in `segmeter` (using the --tool option).

AIList

`AIList v0.1.1` is utilized and applied on unsorted data using the following command:

```
ailist <reference.bed> <query.bed>
```

However, the output is de-duplicated for overlapping intervals in complex queries. In the fourth column, the number of overlaps is reported, which is then used by `segmeter` to ensure that each overlap is represented individually.

BEDops

`BEDops v2.4.41` is utilized, which requires sorted input. Here, we use different commands for basic and complex queries.

```
sort -k1,1 -k2,2n -k3,3n <query.bed> > <query.sorted.bed> # sort the data
bedops --element-of 1 <reference.sorted.bed> <query.sorted.bed> # query basic intervals
bedmap --echo-map --multidelim '\n' <query.sorted.bed> <reference.sorted.bed> # query complex intervals
```

It is to be noted that in `segmeter`, internally the command `bedops` uses the above configuration, while `bedmaps` uses `bedmap` for both basic and complex queries.

bedtools

`bedtools v2.30.0` is utilized, and we applied it to unsorted, sorted, and indexed data:

bedtools (unsorted): Intersection without preprocessing:

```
bedtools intersect -wa -a <reference.bed> -b <query.bed>
```

bedtools with sorted data: Requires pre-sorting of both files:

```
sort -k1,1 -k2,2n -k3,3n <query.bed> > <query.sorted.bed>
bedtools intersect -wa -a <reference.sorted.bed> -b <query.sorted.bed>
```

bedtools with tabix: Uses sorted and indexed data:

```
sort -k1,1 -k2,2n -k3,3n <query.bed> > <query.sorted.bed>
bedtools intersect -wa -a <reference.sorted.bed> -b <query.bed>
```

bedtk

`bedtk` is utilized and applied to both sorted and unsorted. However, `bedtk` has no official release. Hence, we used the version downloaded on 2025-01-25. We used the following command to query basic and complex interval queries:

```
bedtk flt <query.bed> <reference.bed>
```

© The Author 2022. Published by Oxford University Press. All rights reserved. For permissions, please e-mail: journals.permissions@oup.com

However, **bedtk** only reports direct intersections and does not report the original reference intervals (as required for `segmeter`). For that reason, we subsequently applied `bedtools`, which allows us to report the reference intervals from the output of `bedtk`:

```
bedtools intersect -wa -a <filtered.bed> -b <query.bed>
```

However, this was not accounted for in the runtime and memory measurements.

gia

We utilized `gia v0.2.23` and applied it to unsorted data:

```
gia intersect -a <query.bed> -b <reference.bed> -t
```

giggle

We encountered several challenges while working with `giggle v0.6.3`:

1. Compilation issues on Ubuntu $\geq$ 20.04 which has been documented in Issue 9 (https://github.com/ryanlayer/giggle/issues/69)
2. The `giggle index` subcall failed to recognize input and output folders sharing the same parent directory

We created a Docker container (yanglabinfo/segmeter:giggle-latest) based on Ubuntu 20.04 (1), forked `giggle` and modified its data handling implementation (fork available at https://github.com/riasc/giggle). In the working implementation, `segmeter` uses internally the following commands:

```
giggle index -i <indexbedfile> -o <indexfolder> -f -s # create the index
giggle search -i <indexfolder> -q <querybedfile> -v # search for overlap against the index
```

granges

We utilized `granges`, which requires a `--genome` file with the length of each chromosome:

```
granges filter --genome <chromlens_file> --left <reference.bed> --right <query.bed>
```

IGD

We utilized `IGD v0.1.1` and applied it to unsorted data using the following commands:

```
igd create <input_dir> <output_dir> <label> # create index
igd search <index_dir>/<label>.igd -q <query.bed> -f # query intervals
```

However, the output of `IGD` is not in valid BED format and instead reports the overlaps for each query. For that reason, `segmeter` processes the output and converts it into valid BED format. Again, we did account for this in the runtime and memory measurements.

tabix

We utilized `tabix v1.16`. The data needs to be sorted and indexed which was done using the following commands:

```
sort -k1,1 -k2,2n -k3,3n <input.bed> > <sorted.bed> # sort the reference data
bgzip -f <sorted.bed> > <sorted.bed.gz> # compress the data
tabix -f -C -p bed <sorted.bed.gz> # index the data
```

Subsequently, we can query the index reference data using:

```
tabix <sorted.bed.gz> -R <query.bed>
```

UCSC Utils

We used `UCSC utils` from the UCSC utilities (http://hgdownload.soe.ucsc.edu/admin/exe/) and was used in the following command:

```
bedIntersect -aHitAny <input.bed> <query.bed>
```

Similarly, to `bedtk`, we used `bedtools` to report all overlaps.

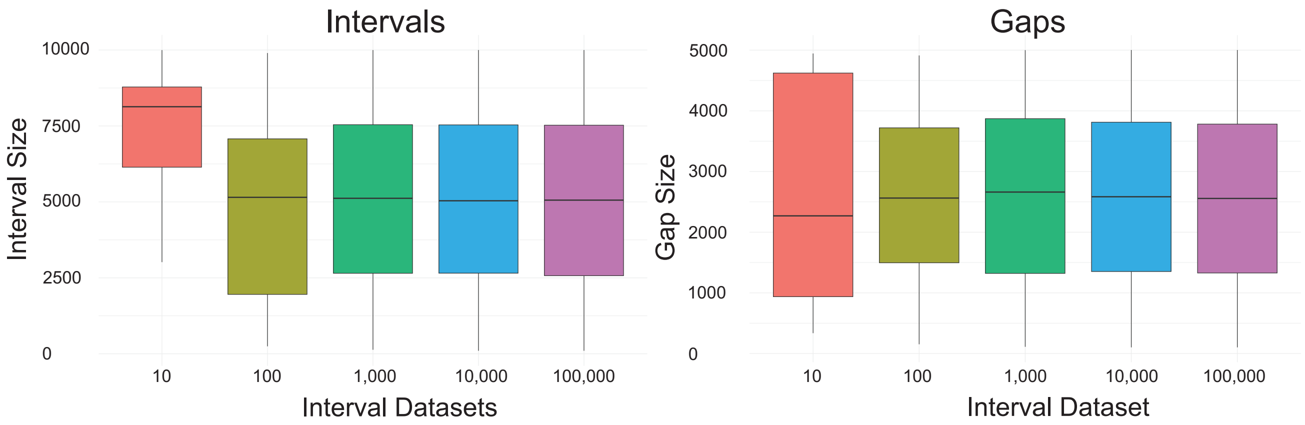

Fig. 1. Interval and gap sizes of the generated interval data.

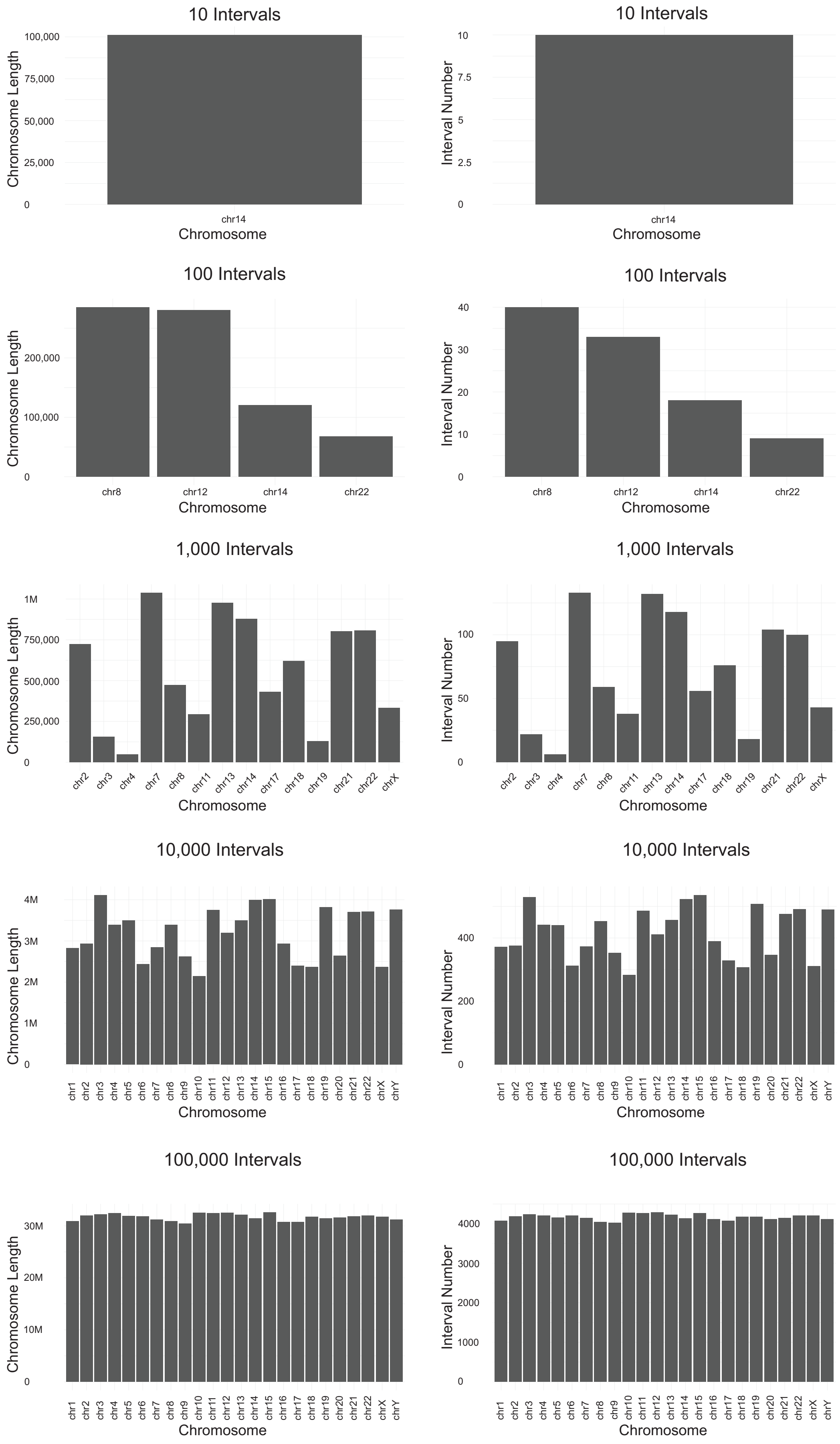

Fig. 2. Chromosome length and interval per chromosome distribution of the simulated datasets

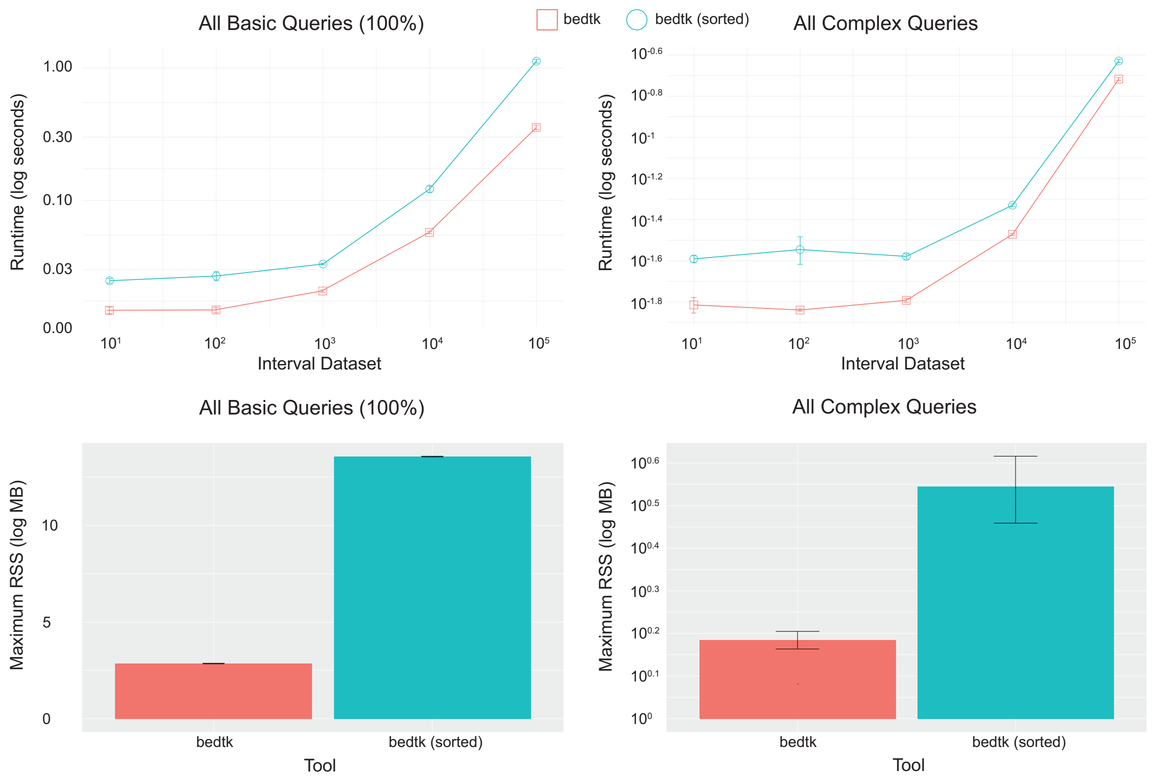

Fig. 3. Runtime memory requirements for `bedtk` compared to `bedtk` on sorted data

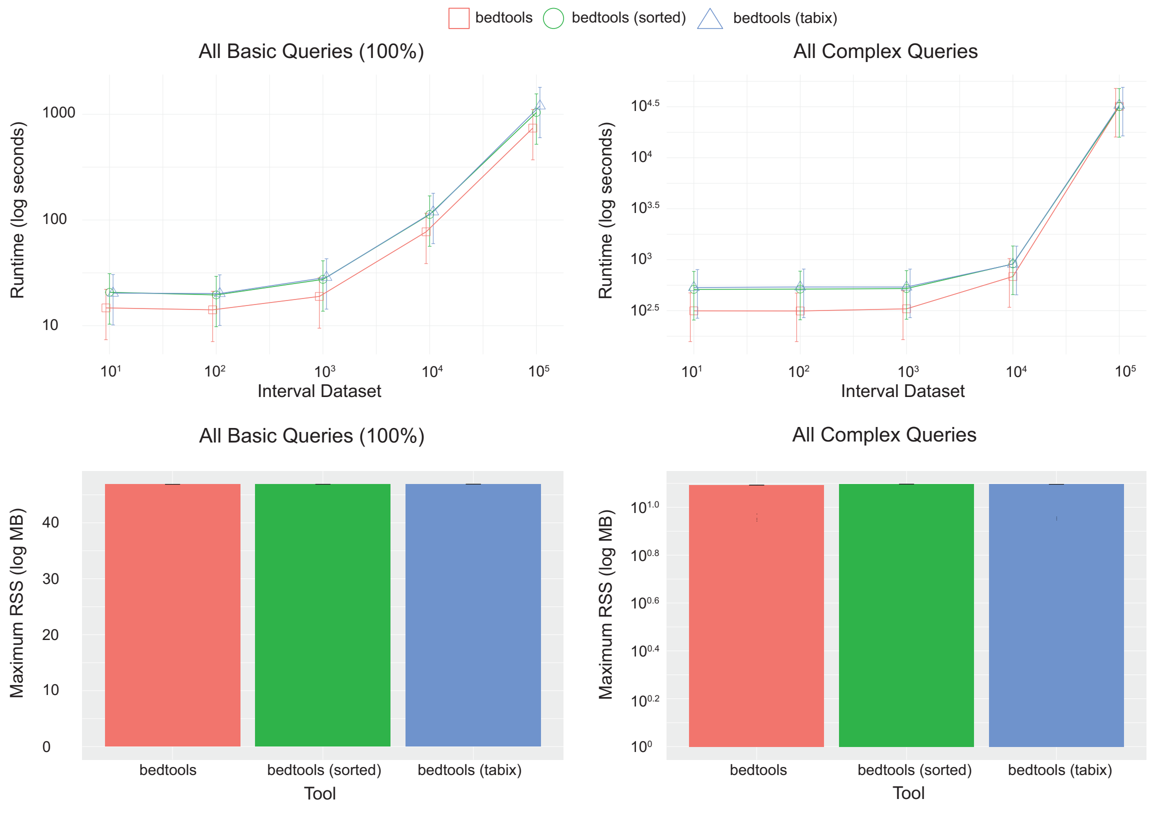

Fig. 4. Runtime and memory requirements for `bedtools` compared to sorted data, and with random access by `tabix`

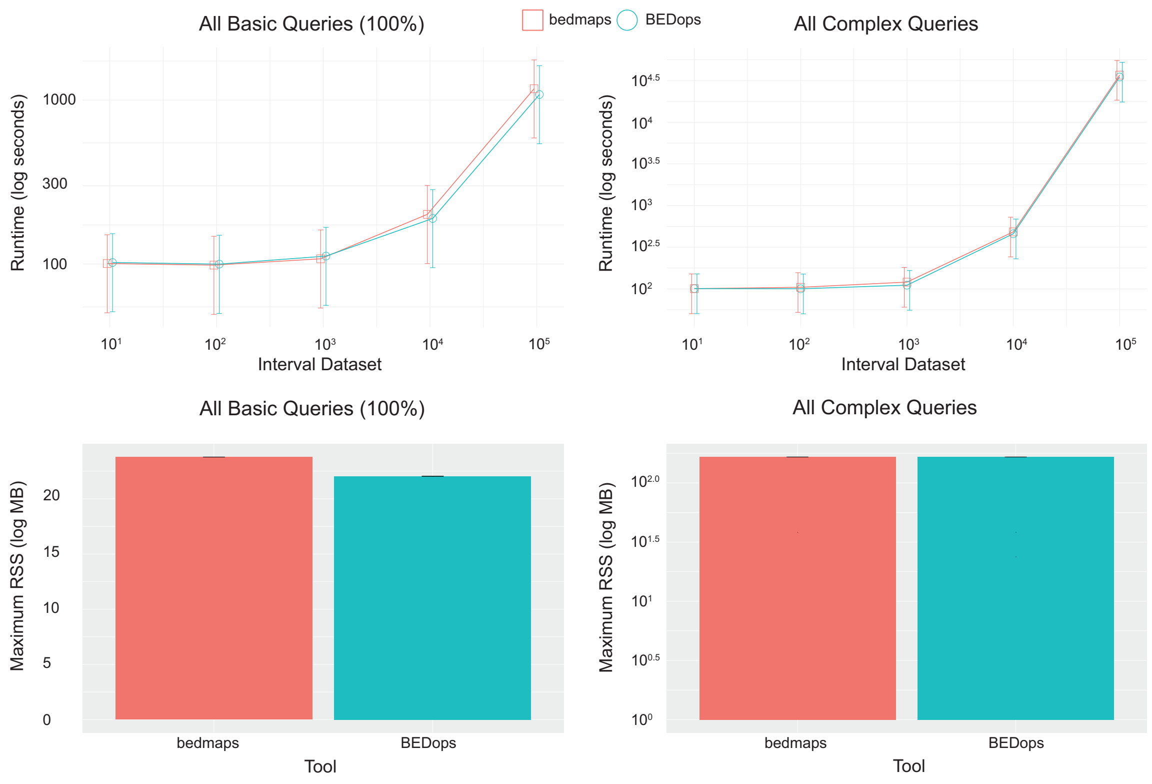

Fig. 5. Comparison of the runtime and memory requirements for `BEDops` and `bedmap` from the `BEDops` utilities in basic and complex queries
